# Review: Multiple system atrophy: emerging targets for interventional therapies

**DOI:** 10.1111/nan.12304

**Published:** 2016-02-29

**Authors:** N. Stefanova, G. K. Wenning

**Affiliations:** ^1^ Division of NeurobiologyDepartment of NeurologyMedical University of InnsbruckInnsbruckAustria

**Keywords:** α‐synuclein, aetiology, clinical trial, multiple system atrophy, neuropathology, pathogenesis

## Abstract

Multiple system atrophy (MSA) is a fatal orphan neurodegenerative disorder that manifests with rapidly progressive autonomic and motor dysfunction. The disease is characterized by the accumulation of α‐synuclein fibrils in oligodendrocytes that form glial cytoplasmic inclusions, a neuropathological hallmark and central player in the pathogenesis of MSA. Here, we summarize the current knowledge on the etiopathogenesis and neuropathology of MSA. We discuss the role of α‐synuclein pathology, microglial activation, oligodendroglial dysfunction and putative cell death mechanisms as candidate therapeutic targets in MSA.

## Introduction

Multiple system atrophy (MSA) is a rare neurodegenerative disorder that presents with a variable combination of autonomic, cerebellar, parkinsonian and pyramidal features 1. Depending on the predominant motor phenotype the disease is clinically sub‐classified in parkinsonian (MSA‐P) or cerebellar (MSA‐C) variant. MSA is an orphan disease with an incidence of up to 2.4 cases per 100 000 persons per year 2, while the prevalence may reach up to 7.8 patients per 100 000 population over the age of 40 3_._ The MSA‐P variant seems to be more common in the western hemisphere 4, 5, whereas MSA‐C appears to be more frequent in Asia 6. The motor symptom onset is usually in the fifth or sixth decade of life 5, 7. However, non‐motor features including cardiovascular autonomic failure, urogenital dysfunction, or respiratory and sleep disorders may precede the motor presentation by some years 7, 8, 9. Cognitive impairment with frontal lobe dysfunction and depression seem to be more common than originally considered 10, 11. The disease duration after clinical diagnosis is usually up to 9 years and disability milestones are reached much earlier as compared to Parkinson's disease (PD) 8.

## Aetiology

Multiple system atrophy is a predominantly sporadic disorder. Genetic studies provide controversial data possibly related to geographical and intra‐group heterogeneity. A recent genome‐wide estimate in 907 MSA pooled cases and 3866 controls defined MSA heritability at 2.09–6.65% which was explained by the presence of misdiagnosed cases in the analysed subgroups 12. Genetic mutations of the COQ2 gene have been linked to MSA as identified in Japanese families 13, but the link between the COQ2 gene and MSA risk was not confirmed in other patient populations 14, 15, 16. Recently, Gaucher disease‐causing GBA variants were associated with MSA 17. Similarly, SNCA polymorphism was proposed to be associated with increased risk for MSA 18, 19, but not confirmed in different patient cohorts 20, 21, 22. Growing, but as yet sparse, evidence supports the notion that epigenetic factors may play a role in MSA 23. Dysregulation of miR‐202 and miR‐96 was associated with MSA 24, 25. Circulating miRNAs were identified to be differentially expressed in MSA patients 26. Finally, environmental toxins were associated with the risk of developing MSA in a few limited epidemiological studies 27, 28. An occupational history of farming was significantly related to higher MSA risk, whereas history of smoking was less common in MSA patients 28.

In summary, all studies on the aetiology of MSA suffer from the limited number of cases as the disease is rare and underdiagnosed. Application of the revised consensus criteria 7 increases diagnostic accuracy but sensitivity remains limited, particularly at first neurological visit. In addition, pathologically proven PD, dementia with Lewy bodies (DLB) and progressive supranuclear palsy may mimick the presentation of MSA, reducing diagnostic accuracy 29. The definite diagnosis of MSA can currently be only made at *post mortem* examination. However, that is missing in the majority of the cases involved in genetic or epidemiological studies, which may contribute to their major limitations and inconclusive results.

## Neuropathology

The pathological diagnostic hallmark of MSA is the ectopic aggregation of α‐synuclein in the cytoplasm of oligodendrocytes, forming wide‐spread glial cytoplasmic inclusions (GCIs) 30, 31, 32. GCIs are typical for MSA and do not seem to commonly occur in the brains of patients with other synucleinopathies like PD or DLB 33. However, neuronal cytoplasmic inclusions of α‐synuclein may be also identified in MSA, but have different characteristics from the classical LBs seen in PD and DLB 34. Furthermore, α‐synuclein aggregation may be found intranuclearly in oligodendrocytes and neurons of MSA brains, but these inclusions seem to be less common than GCIs. 35 The structure of GCIs consists primarily of loosely packed α‐synuclein fibrils 36, but multiple other components can be detected as well 37.

Parallel to the inclusion pathology, distinctive patterns of neuronal loss can be observed in MSA brains. Striatonigral degeneration (SND) underlies MSA‐P and is characterized by loss of dopaminergic neurons in the substantia nigra pars compacta (SNc) as well as projection medium‐spiny neurons GABAergic neurons in the caudate‐putamen. The symptomatology of MSA‐C is linked to olivopontocerebellar atrophy (OPCA) which is characterized by loss of Purkinje neurons in the cerebellar cortex (along with preservation of the neurons in the deep cerebellar nuclei), as well as loss of neurons in the pontine nuclei and the inferior olivary complex 35, 38, 39. Recent stereological analysis indicated neocortical neuronal loss that might underlie cognitive impairment in MSA cases 40. Non‐motor symptoms are strongly associated with neurodegeneration in the brainstem and spinal cord. Typically affected regions are the locus ceruleus, the ventrolateral tegmental nucleus, the pedunculopontine tegmental area 41, 42, 43, catecholaminergic neurons of the rostral ventrolateral medulla (C1 group) and noradrenergic neurons of the caudal ventrolateral medulla (A1 group) 44, neurons in the pontine micturition area 45, serotonergic neurons in the nucleus raphe magnus, raphe obscurus, raphe pallidus and ventrolateral medulla 46, as well as neuronal loss in the dorsal vagal nucleus, the ventrolateral nucleus ambiguus 47 and the periaqueductal grey 43, 48. Spinal cord pathology is characterized by neuronal loss in the intermediolateral columns and the Onuf's nucleus in the lumbosacral region 49, 50. Recent high‐definition optical coherence tomography analysis suggested progressive retinal changes in MSA patients with reduction in the thickness of the retinal nerve fibre layer and in the macular ganglion cell complex 51.

Demyelination with variable severity is observed in MSA white matter, but severe myelin lesions seem to be present in only 50% of cases, predominantly in the MSA‐C variant 52. Myelin proteins including sphingomyelin, sulfatide and galactosylceramide were reported decreased by about 50% in degenerating MSA white matter 53.

Gliosis is invariably described in the degenerating areas of the MSA brain 54. Region specific astroglial activation was reported to positively correlate with the α‐synuclein pathology in MSA cases in contrast to PD 55. Microglial activation is prominent in the degenerating regions of MSA brains and accompanies GCI pathology 52, 56. Upregulation of TLR4 57 and myeloperoxidase 58 has been shown in activated MSA microglia.

The peripheral nervous system and the autonomic nervous system in MSA have been the focus of studies recently, but the outcomes are still controversial and under debate. In skin biopsies phosphorylated α‐synuclein accumulation was shown in unmyelinated somatosensory fibres of 67% of MSA patients, whereas in a PD cohort phosphorylated α‐synuclein seemed to accumulate in autonomic fibres 59. However, other studies indicate lack of phosphorylated α‐synuclein immunoreactivity in dermal nerve fibres in MSA in contrast to PD patients 60, 61. Importantly, recent work showed α‐synuclein pathology affecting Schwann cells of cranial, spinal and autonomic nerves in MSA cases 62. Furthermore, α‐synuclein pathology in the enteric nervous system of MSA patients may occur 63.

## Pathogenesis

The pathogenesis of MSA remains largely unknown. It is currently accepted that the oligodendroglial α‐synuclein accumulation plays a central role in the disease process. A correlation between the GCI load and the degree of neuronal loss was reported in both the striatonigral and the olivopontocerebellar regions 39. In the white matter, both GCI burden as well as microglial activation were shown to be greater in tissue with mild to moderate demyelination, and to decrease when demyelination became severe 52. The leading role of GCI pathology in MSA was further supported by cases of so‐called minimal change MSA. In such cases severe GCI burden associated with less severe neuronal loss triggered rapidly progressive clinical MSA profile at a younger age, with significantly shorter duration as compared to ‘classical’ MSA cases 64.

Neuronal α‐synuclein‐positive cytoplasmic inclusions (NCIs) seem to be much more widespread than previously assumed 34. However, NCIs can be composed of non‐fibrillar α‐synuclein and show hierarchical pattern of neuronal involvement related with the duration of the disease, but rather independent of the pattern of neuronal destruction suggesting that other factors play a leading role in the subtype‐dependent neuronal loss 34. Although it is tempting to speculate that primary neuronal pathology leads to secondary oligodendroglial α‐synuclein accumulation as suggested by the finding that NCIs may exist in areas that lack GCIs 34, the robust observation that distribution and severity of neurodegeneration reflect subregional GCI densities supports the hypothesis of a primary oligodendrogliopathy.

The causative role of GCI‐like pathology for the induction of neuronal loss was confirmed experimentally in transgenic mice overexpressing human α‐synuclein in oligodendrocytes under various oligodendroglia‐specific promoters 65, 66, 67, 68, 69. The selectivity of neurodegeneration in these models as well as in the human disease is still unresolved. In the 2,′3′‐cyclic nucleotide 3′‐phosphodiesterase (CNP)‐α‐synuclein mouse the neuronal loss related to the GCI pathology mostly affected cortical and spinal cord regions linked to secondary axonal degeneration 65. When overexpressed under the myelin basic protein (MBP) promoter, α‐synuclein in oligodendrocytes triggered dose‐dependent neuronal loss in the neocortex, and fibre degeneration in the basal ganglia without loss of nigral neurons as well as demyelination and astrogliosis in the white matter tracts 66. The proteolipid (PLP)‐α‐synuclein transgenic mouse 67 modelled to a great extent 70 the specific neuropathology of MSA, including progressive nigrostriatal neuronal loss 68, 71 as well as loss of neurons in autonomic centres relevant to the human disease 69, 72, 73. The PLP‐α‐synuclein transgenic mouse is the only one that replicates microglial activation accompanying the neurodegeneration of MSA type 57. Whether the specificity of distribution of the promoters used (CNP *vs*. MBP *vs*. PLP) or the triggering of different disease cascades, that is (i) GCIs and secondary axonal degeneration in the CNP‐α‐synuclein model resulting in spinal cord and cortical degeneration; (ii) GCIs and secondary demyelination and astrogliosis in the MBP‐α‐synuclein model resulting in neocortical degeneration or (iii) GCIs and microglial activation resulting in nigral neuronal loss and degeneration in autonomic centres of PLP‐α‐synuclein mice is under debate. Intriguingly the presence of GCIs and microglial activation in PLP‐α‐synuclein mice makes them more susceptible to exogenous oxidative or proteolytic stress and moreover triggers MSA‐like selective SND and OPCA not observed in wild‐type mice exposed to the same stress factors 68, 74. In conclusion, it seems that the selectivity of neurodegeneration in MSA is determined by the concerted interaction of multiple factors, among them the ectopic α‐synuclein accumulation in oligodendrocytes, microglial activation, oxidative stress and proteolytic dysbalance.

**Figure 1:**
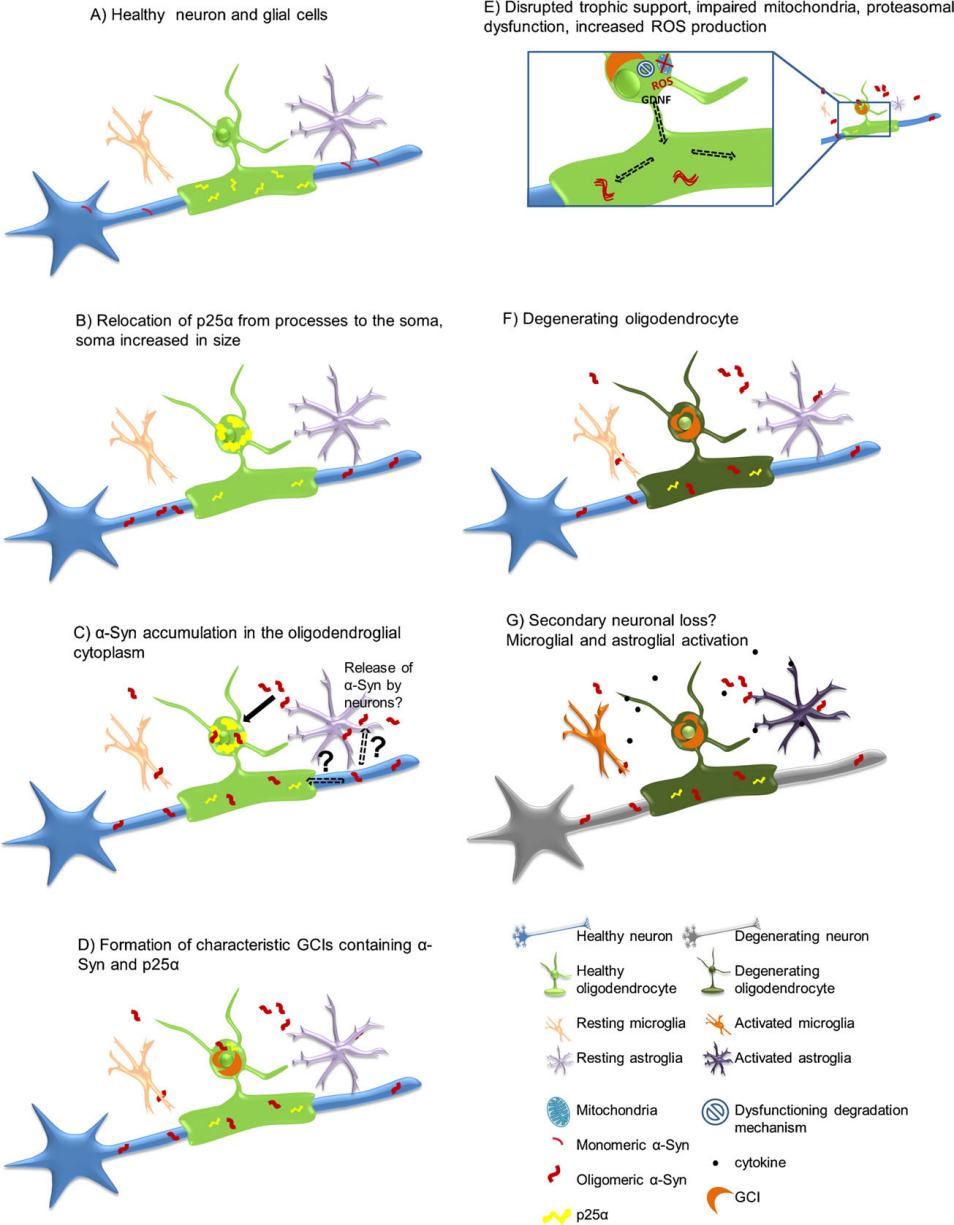
Possible pathological α‐synuclein‐spreading and accumulation mechanism leading to neurodegeneration. (**A**) Healthy neuron, oligodendrocyte, microglia and astrocyte, p25α mainly located in the myelinating oligodendroglial processes, monomeric α‐synuclein present in presynaptic nerve terminals. (**B**) Relocalization of p25α from the processes to the soma, inclusion formation and swelling of the oligodendroglial soma. (**C**) Oligomeric α‐synuclein accumulation in the oligodendroglial cytoplasm‐(the exact source of α‐synuclein remains to be determined). Possible hypotheses include exocytosed α‐synuclein from neurons and uptake into oligodendrocytes by cell‐to‐cell propagation or upregulation of α‐synuclein expression in oligodendrocytes themselves. In addition, axonal α‐synuclein may be taken up by the dysfunctional oligodendroglial myelin compartment. (**D**) α‐synuclein aggregates form insoluble half‐moon shaped glial cytoplasmic inclusions (GCIs) characteristic for the disease. (**E**) Disruption of trophic support (e.g. GDNF), mitochondrial failure, increased production of reactive oxygen species (ROS) and proteasomal dysfunction. (**F**) Oligodendrocytes suffer from severe distress and will eventually degrade. (**G**) Activation of micro/astroglial cells by cytokines released from the damaged oligodendrocytes, and proposed secondary neuronal loss potentially due to lack of trophic support, ROS production, proteasomal failure and pro‐inflammatory environment. Reproduced from Kuzdas‐Wood *et al*. 106, doi:10.1016/j.pneurobio.2014.02.007, available under the terms of the Creative Commons Attribution License (CC BY).

The source of α‐synuclein in GCIs is debatable. Earlier studies suggested that α‐synuclein is an exclusively neuronal protein that is not expressed in healthy adult oligodendroglia 75, 76, 77. These data suggested that the source of α‐synuclein that accumulated in MSA oligodendroglia was the diseased neurons (Figure 1). In light of the recent findings on α‐synuclein cell‐to‐cell transmission, this hypothesis became more plausible 78. Prusiner and co‐workers suggested that MSA‐ and not PD‐derived α‐synuclein may induce α‐synuclein‐positive inclusion propagation in a transgenic model of PD 79, 80. However, the same studies failed to provide evidence for propagation of MSA‐derived α‐synuclein inclusions in wild‐type/healthy mouse brain and furthermore no oligodendroglial α‐synuclein aggregation was reported, therefore failing to reproduce the core pathology of MSA. Experimental studies showed that oligodendrocytes may take up α‐synuclein from the extracellular space 81, but in none of these cases was typical GCI‐like aggregation reported. It seems that healthy oligodendrocytes are normally able to cope with the uptaken α‐synuclein and successfully ‘digest’ it without forming GCIs. Primary oligodendroglial dysfunction may be therefore result in ectopic accumulation of α‐synuclein in oligodendrocytes 82. Alternatively, specific α‐synuclein conformational strains were proposed to be responsible for the generation of PD‐ and MSA‐like α‐synuclein seeding traits 83. However, this hypothesis needs further clarification and support, as inoculation of MSA‐derived α‐synuclein into the brains of healthy non‐transgenic mice did not induce GCI‐like pathology, nor did. PD‐derived α‐synuclein show prion‐like behaviour in any of the models (transgenic or non‐transgenic) used 80. In summary, the existing data indicate that neuron‐derived α‐synuclein with certain conformational changes may contribute to the formation of GCIs, and that primary oligodendroglial dysfunction of as yet unknown origin is permissive and may be obligatory for α‐synuclein fibrils to accumulate in the cytoplasm of oligodendrocytes in MSA. In support of the notion of a primary oligodendrogliopathy p25α/TPPP dislocation and accumulation in MSA oligodendroglial soma were shown to precede the accumulation of α‐synuclein 84 (Figure 1).

Recent studies based on modern technologies like laser dissection and iPSCs technology have cast doubt upon the classical dogma that α‐synuclein or SNCA mRNA are not expressed by oligodendrocytes. Asi *et al*. 85 showed that laser‐dissected oligodendrocytes from healthy or MSA brains do show α‐synuclein expression, and even identified a tendency to increase in the signals in MSA oligodendrocytes. Djelloul *et al*. 86 proposed that oligodendrocytes differentiated from MSA‐derived iPSCs express α‐synuclein in contrast to those derived from healthy controls or PD patients. Although having some methodological limitations, these studies raise the possibility that an endogenous intra‐oligodendroglial α‐synuclein source may contribute to the GCI formation in MSA, and thus play a primary role in triggering MSA neurodegeneration.

As already mentioned, microglial activation is a prominent finding in the degenerating brain areas in MSA, and can be visualized both neuropathologically 56 as well as by *in vivo* PET imaging 87. The association of activated microglial cells and GCI burden 56 suggests that pathological α‐synuclein triggers neuroinflammatory responses in the MSA brain. This hypothesis was corroborated by a number of experimental studies both *in vitro* and *in vivo* 54, 88, 89. Furthermore, observations in PLP‐α‐synuclein transgenic mice suggested that microglial activation occurred parallel to the dopaminergic neuronal loss in SNc between the age of 2 and 4 months. These changes could be reversed by suppression of microglial activation using minocycline 57. TLR4 dysfunction in microglia of PLP‐α‐synuclein transgenic mice led to accumulation of α‐synuclein in the brains and resulted in aggravated functional phenotype and increased nigral neuronal loss, suggesting that a subpopulation of microglia might play an important role in the clearance of α‐synuclein and neuroprotection 88. In summary, compelling evidence supports the notion that microglial activation may contribute to the progression of the degenerative process in MSA, like in other neurodegenerative diseases 90, 91, and although non‐specific this mechanism may be exploited for therapeutic interventions.

Oligodendroglial dysfunction may also be a primary event in MSA pathogenesis 82, but it is plausible that the accumulation of α‐synuclein in oligodendrocytes may deepen and broaden this dysfunction resulting in reduced trophic support and demyelination, as suggested by findings in the MBP‐α‐synuclein transgenic mouse model of MSA 66, 92. Furthermore, changes in sphingomyelin, sulfatide and galactosamide levels in MSA brains suggested myelin lipid dysfunction and instability 53, 93. The oligodendroglial precursor cells are responsible for remyelination and show increased density in MSA white matter suggesting disease‐associated repair efforts. 94. Taken together the data on oligodendroglial dysfunction in MSA support the possible scenario that neurodegeneration may occur secondarily to the demyelination and lack of trophic support by oligodendrocytes bearing GCIs.

Finally, the cell death mechanisms in MSA are to a great extent unresolved. Early studies suggested increased iron levels in the degenerating areas 95, supporting the notion that oxidative stress might play a significant role in the selective neuronal loss in MSA. This idea was strongly supported by the report on COQ2 mutations linked to mitochondrial dysfunction in MSA cases 13. Microglial activation might contribute to the increased levels of reactive oxygen species in the degenerating areas 58. In addition to oxidative stress α‐synuclein oligomers and fibrils might directly exert neurotoxicity. Experimental studies demonstrated that α‐synuclein oligomers might disintegrate the cellular membranes by forming pores 96, however, this hypothesis was never proven in MSA. Alternatively α‐synuclein fibril accumulation in cells might induce metabolic imbalance which might in turn promote cell death. Intriguingly, exclusive oligodendroglial apoptosis was reported in MSA brains 97, 98. Phosphoinositide 3‐kinase upregulation was found in neurons and oligodendrocytes in MSA, suggesting a possible response to apoptotic signals in these cells 99. Furthermore, the X‐linked inhibitor of apoptosis protein, which selectively binds to caspases‐3, ‐7 and ‐9, and inhibits their activities, was found to be upregulated in GCI‐ and NCI‐bearing oligodendrocytes and neurons respectively 100. The expression of the calcium binding proteins calbindin and parvalbumin in Purkinje cells was found to be significantly reduced in MSA, whereas the apoptosis modulating proteins Bax, and Bcl‐x were increased, suggesting that a diminished calcium binding capacity might lead to the pathological initiation of apoptosis in the affected areas 98. Other mechanisms that have been discussed to relate to the cell death in MSA include proteasomal 101 or autophagosomal dysfunction 102 and excitotoxicity 103. While experimental studies support the involvement of the proteasome and autophagosome dysfunction in oligodendroglial α‐synucleinpathy 74, 104, excitotoxic cell death was not aggravated by GCI pathology 105.

## Target validation – where are we now?

Although the exact sequence of events in the pathogenesis of MSA is still hypothetical, there are several main players that definitely contribute to the disease process and its progression and may serve as prominent targets for disease therapy. These include (i) pathological α‐synuclein species accumulation, (ii) microglial activation and neuroinflammatory responses, (iii) oligodendroglial dysfunction and (iv) cell death (Table 1). Most of the preclinical screening studies in α‐synuclein transgenic models confirm the feasibility of the above‐mentioned targets for the treatment of MSA. However, in a clinical setting the same therapeutic approaches appeared insufficient to provide disease modification and reduce the progression of symptoms. A major difference between the preclinical studies and the clinical trials relates to the disease stage when the therapies are initiated. The clinical diagnosis of MSA is possible only after the motor symptoms become overt 7. This disease stage may represent a rather late stage of the pathogenic process in MSA with a significant degree of neuronal loss that is hardly reversible. As it is likely that the disease onset precedes the motor presentation by many years, identification of early biomarkers to determine at risk individuals in selected cohorts of patients with neurogenic orthostatic hypotension, urogenital dysfunction or REM sleep behaviour disorder may significantly improve the outcomes in the treatment of MSA.

**Table 1:** Experimental therapies for MSA

Therapy	Target(s)	α‐Synuclein MSA model	Efficacy in α‐Synuclein MSA model(s)	Clinical trial
Recombinant human growth hormone	Neuronal and glial proliferation	n.a.	n.a.	**Design:** Randomized, double‐blind, placebo‐controlled; **Primary end‐point:** UPDRS (Unified Parkinson's Disease Rating Scale)‐total, autonomic testing; **Result:** Ineffective 107
Minocycline	Microglial activation	PLP‐α‐synuclein 57	Suppression of microglial activation, neuroprotection in early disease stage	**Design:** Randomized, double‐blind, placebo‐controlled; phase III study **Primary end‐point:** Change of UMSARS (Unified Multiple System Atrophy Rating Scale)‐II **Result:** Suppression of microglial activation, but no change of symptom severity 108
Riluzole	Cell death	n.a.	n.a.	**Design:** Randomized, double‐blind, placebo‐controlled; phase III study **Primary end‐point:** survival **Result:** No effect on survival and motor decline 109
Autologous MSCs (intravenous and intraarterial)	Unclear	PLP‐α‐synuclein 110	Modulation of neuroinflammatory responses; mild nigral neuroprotection	**Design:** Randomized, double‐blind, placebo‐controlled; phase II study **Primary end‐point:** Change of UMSARS‐total **Result:** Slowed disease progression 111, safety concerns
Lithium	α‐synuclein	n.a.	n.a.	**Design:** Randomized, double‐blind, placebo‐controlled; phase II study **Primary end‐point:** frequency of severe AE **Result:** Discontinued due to safety concerns 112
Rifampicin	α‐synuclein	MBP‐α‐synuclein 113	Reduced α‐synuclein load, reduced astrogliosis, neuroprotection	**Design:** Randomized, double‐blind, placebo‐controlled; phase III study **Primary end‐point:** UMSARS, MR parameters, BDI‐II, EQ‐5D scale **Result:** Study terminated, ineffective 114
Rasagiline	Cell death	PLP‐α‐synuclein + 3NP 115	Neuroprotection and functional improvement in early disease stage	**Design:** Randomized, double‐blind, placebo‐controlled; phase II study **Primary end‐point:** Change of UMSARS‐total **Result:** No change in outcome measures 116
Fluoxetine	Neuroinflammation, Oligodendroglial dysfunction	MBP‐α‐synuclein 117	Modulation of trophic factor support, improved neurogenesis, reduced astrogliosis, ameliorated demyelination, reduced α‐synuclein aggregation, neuroptotection and functional improvement	**Design:** Randomized, double‐blind, placebo‐controlled; phase II study **Primary end‐point:** Change of UMSARS‐II **Result:** pending
MPO inhibitor (AZD3241)	Microglial activation	PLP‐α‐synuclein + 3NP 58, 118	Suppression of microglial activation, reduced α‐synuclein aggregation, neuroprotection and functional improvement in early disease stage Suppression of microglial activation but no neuroprotection nor functional improvement in advanced disease	**Design:** Randomized, double‐blind, parallel group; phase II study **Primary end‐point:** safety, tolerability and effect on microglia activation via PET imaging **Result:** Ongoing
Active immunization (AFFITOPE PD01A; PD03A)	α‐synuclein	MBP‐α‐synuclein 119	Reduced α‐synuclein load, reduced demyelination, neuroprotection, functional improvement	**Design:** A randomized, placebo‐controlled, parallel group, patient‐blind, phase I study; **Primary end‐point:** Safety and immunological/therapeutic activity **Result:** Ongoing
Autologous MSCs (intrathecal)	Unclear	PLP‐α‐synuclein 110	Modulation of neuroinflammatory responses; mild nigral neuroprotection	**Design:** Open label; Single group assignment, phase I study **Primary end‐point:** Safety/Efficacy **Result:** Ongoing

MSA, multiple system atrophy; 3NP, 3‐nitropropionic acid; MBP, myelin basic protein; MPO, myeloperoxidase, MSCs mesenchymal stem cells; PLP, proteolipid protein; AE, adverse events.

In summary, future studies on MSA will need to focus on better understanding of the triggers of the disease process, as well as on improved diagnosis, with identification of early biomarkers that may allow the timely initiation of disease modifying therapies with beneficial effects on disease progression.
